# Er-Doped LiNi_0.5_Mn_1.5_O_4_ Cathode Material with Enhanced Cycling Stability for Lithium-Ion Batteries

**DOI:** 10.3390/ma10080859

**Published:** 2017-07-27

**Authors:** Shanshan Liu, Hongyuan Zhao, Ming Tan, Youzuo Hu, Xiaohui Shu, Meiling Zhang, Bing Chen, Xingquan Liu

**Affiliations:** 1 R&D Center for New Energy Materials and Devices, State Key Laboratory of Electronic Thin Film and Integrated Devices, University of Electronic Science and Technology of China, Chengdu 610054, China; shanshannay@163.com (S.L.); mingtan_kilu@126.com (M.T.); huyouzuo@126.com (Y.H.); shu_xh2016@163.com (X.S.); zhangmeiling199310@163.com (M.Z.); 2 Xinyun Electronic Comp. & Dev. Co. Ltd, China Zhenhua Group, Guiyang 550018, China

**Keywords:** Lithium-ion battery, LiNi_0.5_Mn_1.5_O_4_, Er-doping, citric acid-assisted sol-gel method, cycling stability

## Abstract

The Er-doped LiNi_0.5_Mn_1.5_O_4_ (LiNi_0.495_Mn_1.495_Er_0.01_O_4_) sample was successfully prepared by citric acid-assisted sol-gel method with erbium oxide as an erbium source for the first time. Compared with the undoped sample, the Er-doped LiNi_0.5_Mn_1.5_O_4_ sample maintained the basic spinel structure, suggesting that the substitution of Er^3+^ ions for partial nickel and manganese ions did not change the intrinsic structure of LiNi_0.5_Mn_1.5_O_4_. Moreover, the Er-doped LiNi_0.5_Mn_1.5_O_4_ sample showed better size distribution and regular octahedral morphology. Electrochemical measurements indicated that the Er-doping could have a positive impact on the electrochemical properties. When cycled at 0.5 C, the Er-doped LiNi_0.5_Mn_1.5_O_4_ sample exhibited an initial discharge capacity of 120.6 mAh·g^−1^, and the capacity retention of this sample reached up to 92.9% after 100 cycles. As the charge/discharge rate restored from 2.0 C to 0.2 C, the discharge capacity of this sample still exhibited 123.7 mAh·g^−1^ with excellent recovery rate. Since the bonding energy of Er-O (615 kJ·mol^−1^) was higher than that of Mn-O (402 kJ·mol ^−1^) and Ni-O (392 kJ·mol^−1^), these outstanding performance could be attributed to the increased structure stability as well as the reduced aggregation behavior and small charge transfer resistance of the Er-doped LiNi_0.5_Mn_1.5_O_4_.

## 1. Introduction

Lithium-ion batteries are widely believed to be the most promising power sources for next-generation electrical equipment. As an important component, cathode materials have a large impact on the electrochemical properties of lithium-ion batteries [1,2]. Up to now, researchers have developed several cathode materials, such as LiCoO_2_, LiFePO_4_, LiMn_2_O_4_, and LiNi_x_Co_y_M_z_O_2_ (M = Mn, Al), etc.

Among these commercial materials, LiMn_2_O_4_ has broad development prospects because of the abundant manganese resource and environmental protection performance. However, this material presents poor cycling stability, especially at high temperature. For the derivatives of LiMn_2_O_4_, transition metal doped LiM*_x_*Mn_2−*x*_O_4_ (M = Cu [3], Ni [4], Fe [5], Co [6], Cr [7], etc.) cathode materials can exhibit a high voltage plateau at around 5.0 V, which creates a good condition for increasing the energy density and power density. Among them, LiNi_0.5_Mn_1.5_O_4_ has been recognized as the ideal high-voltage material due to the abundant nickel and manganese resources, eco-friendliness, low-cost, etc. [8,9,10]. As a result, LiNi_0.5_Mn_1.5_O_4_ has attracted much attention from academics and enterprises. According to the existing literatures [11,12], the LiNi_0.5_Mn_1.5_O_4_ structure (disordered or ordered structure) depends on the ordering of nickel and manganese ions. Compared with the ordered sample, the disordered sample can show better electrochemical performance because of excellent electronic conductivity and low activation energy [13,14]. And the actual discharge capacity of this material can achieve about 130 mAh·g^−1^. Therefore, LiNi_0.5_Mn_1.5_O_4_ has great prospect in cathode materials for high-performance lithium-ion batteries. However, the commercial application of LiNi_0.5_Mn_1.5_O_4_ is restricted by some factors, such as the chemical dissolution of Mn, existence of Li*_x_*Ni_1−*x*_O impurities, oxidative decomposition of electrolyte working at high voltage and so on [15,16,17]. According to research results [18,19], doping technique can play an active role in modifying the LiNi_0.5_Mn_1.5_O_4_. Liu et al. [20] have prepared the Er-doped LiMn_2_O_4_ sample by the rheological phase reaction method. The Er-doped LiMn_2_O_4_ can display good cycling stability, indicating that small amounts of Er^3+^ ions can promote the improvement of electrochemical properties. Moreover, the Er-doping also demonstrates a positive role in improving the cycling stability of other cathode materials such as LiFePO_4_ and LiV_3_O_8_ [21,22]. Therefore, it is interesting to speculate that the substitution of Er^3+^ ions for partial nickel and manganese ions may show a positive effect on the electrochemical performance of LiNi_0.5_Mn_1.5_O_4_.

In this work, the Er-doped LiNi_0.5_Mn_1.5_O_4_ was successfully prepared by the citric acid-assisted sol-gel method with erbium oxide as the erbium source for the first time. The substitution of Er^3+^ ions for partial nickel and manganese ions did not change the intrinsic structure of LiNi_0.5_Mn_1.5_O_4_. Moreover, the Er-doped LiNi_0.5_Mn_1.5_O_4_ showed better size distribution and regular octahedral morphology. The effect of doping with Er^3+^ ions on the electrochemical performance of LiNi_0.5_Mn_1.5_O_4_ was studied in detail.

## 2. Experimental

The Er-doped LiNi_0.5_Mn_1.5_O_4_ (LiNi_0.495_Mn_1.495_Er_0.01_O_4_) sample was successfully prepared by citric acid-assisted sol-gel method. Firstly, a certain amount of erbium oxide was suspended in concentrated nitric acid and continuously stirred for 30 mins. And then, the erbium oxide turbid liquid was refluxed at 80 °C until a clear solution was obtained. The pink solution was shifted in a small beaker and evaporated to remove excess nitric acid. Subsequently, stoichiometric amounts of manganese acetate, nickel acetate, and erbium nitrate were dissolved in deionized water. The molar ratio of Li:Ni:Mn:Er:O was 1:0.495:1.495:0.01:4. The mixed solution of citric acid and lithium hydroxide was prepared and added to the former solution under stirring. Next, adding ammonia water manipulated the pH of the obtained solution to about 7.5. The achieved solution was evaporated at 80 °C to get a wet greenish gel. After being dried at 110 °C for 24 h in oven, the greenish gel was sintered at 450 °C for 4 h in air. Subsequently, the decomposed gel precursor was ground in mortar and sintered at 800 °C for 18 h in air to get the darkish product. The undoped LiNi_0.5_Mn_1.5_O_4_ sample was prepared by the same citric acid-assisted sol-gel technology.

In order to confirm the crystal structure, X-ray diffraction (XRD, Bruker DX-1000, Cu Kα radiation, Bruker Corporation, Karlsruhe, Germany) was carried out. The surface morphology of the LiNi_0.5_Mn_1.5_O_4_ and Er-doped LiNi_0.5_Mn_1.5_O_4_ samples was identified by scanning electron microscopy (SEM, Japanese Electronics Co., Ltd, Tokyo, Japan). For electrochemical evaluation, the working electrode was constituted from 85% synthesized product as cathode material, 10% acetylene black as conductive agent, and 5% polyvinylidene fluoride dissolved in N-methyl-2-pyrrolidone as binder. The working electrode was tested by using CR2025 coin-type cells. The electrode mixture was pasted on the aluminum foil and dried at 100 °C for 30 min, and then compressed to obtain round positive plates. Lithium foil and polypropylene membrane were used as anode material and diaphragm, respectively. 1 M LiPF_6_ in a mixture (V_DMC_:V_EMC_:V_EC_ = 1:1:1) was used as the electrolyte. Electrochemical measurements were carried out by using LAND CT2001A (Wuhan LAND Electronics Co., Ltd., Wuhan, China). Electrochemical impedance spectra (EIS) and cyclic voltammogram (CV) were studied by CS-350 electrochemical workstation (Wuhan Corrtest Instruments Corp., Ltd., Wuhan, China).

## 3. Results and Discussion

**Figure 1:**
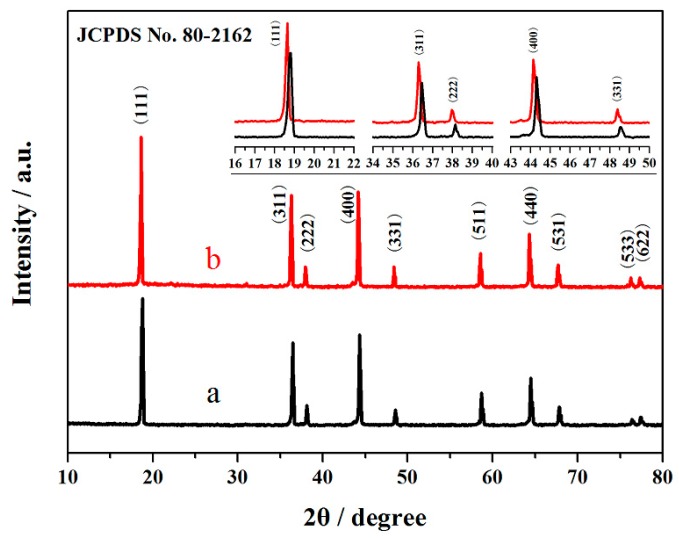
XRD patterns of the LiNi_0.5_Mn_1.5_O_4_ (**a**) and Er-doped LiNi_0.5_Mn_1.5_O_4_ (**b**).

Figure 1 shows the XRD patterns of the LiNi_0.5_Mn_1.5_O_4_ and Er-doped LiNi_0.5_Mn_1.5_O_4_ samples. The characteristic diffraction peaks of the Er-doped LiNi_0.5_Mn_1.5_O_4_ sample are indexed to LiNi_0.5_Mn_1.5_O_4_ (JCPDS No. 80-2162) [23]. This indicates that the substitution of Er^3+^ ions for partial nickel and manganese ions has very little effect on the intrinsic structure [24,25,26,27]. Notably, the diffraction peaks show a small shift to smaller 2θ angle for the Er-doped LiNi_0.5_Mn_1.5_O_4_ sample with the introduction of a certain amount of Er^3+^ ions. This phenomenon can be interpreted as the successful incorporation of Er^3+^ ions into the crystal structure and an increase of the unit cell volume of the Er-doped LiNi_0.5_Mn_1.5_O_4_. These results can be associated with the fact that the radius of Er^3+^ ions (1.04 Å) [20] is bigger than that of Mn^4+^ ions (0.53 Å) [25,26] and Ni ions (0.69 Å) [9]. According to the research results [18,27], if the dopant ions are located on the tetrahedral (8a) sites, the intensity of (220) peak, which arises only from the diffraction of the tetrahedral sites, must increase, even if the doping concentration is very low. From the Figure 1, it can be noted that the (220) peak does not appear in the XRD pattern of Er-doped LiNi_0.5_Mn_1.5_O_4_ sample. This indicates that the tetrahedral sites (8a) are occupied by Li^+^ ions, the Er^3+^ ions only occupy the octahedral (16d) sites to substitute nickel and manganese ions. Furthermore, the diffraction intensities of the Er-doped LiNi_0.5_Mn_1.5_O_4_ are stronger, which suggests that the substitution of Er^3+^ ions for partial nickel and manganese ions can enhance the crystallinity of LiNi_0.5_Mn_1.5_O_4_.

**Figure 2:**
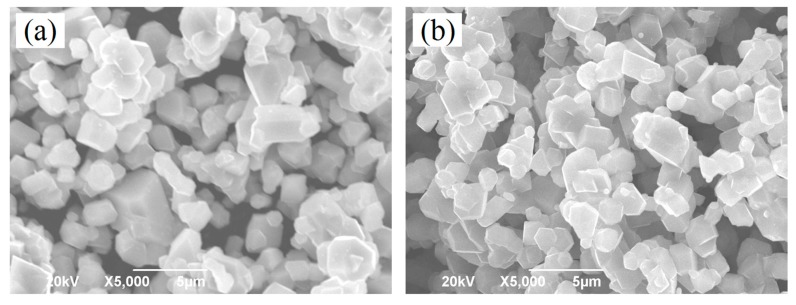
SEM images of the LiNi_0.5_Mn_1.5_O_4_ (**a**) and Er-doped LiNi_0.5_Mn_1.5_O_4_ (**b**).

**Figure 3:**
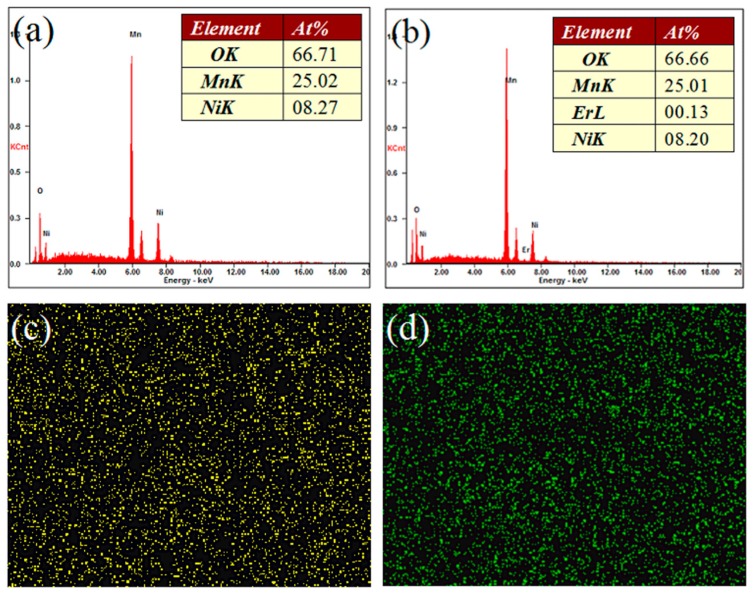

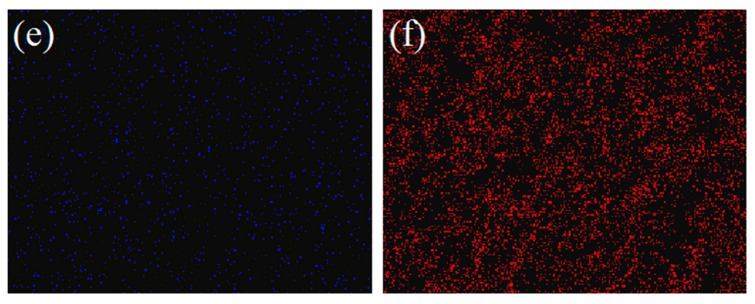
Energy dispersive spectrometry (EDS) patterns of the undoped LiNi_0.5_Mn_1.5_O_4_ (**a**) and Er-doped LiNi_0.5_Mn_1.5_O_4_ (**b**) sample (The inset in Figure 3a,b is corresponding EDS data); (**c**–**f**) Elemental mapping images of Ni, Mn, Er, and O elements in the Er-doped LiNi_0.5_Mn_1.5_O_4_ sample.

Figure 2 shows the SEM images of the LiNi_0.5_Mn_1.5_O_4_ and Er-doped LiNi_0.5_Mn_1.5_O_4_ samples. As shown in Figure 2a, we can see that the undoped LiNi_0.5_Mn_1.5_O_4_ sample presents very undesirable grain size distribution with average particle size distribution of about 2.0 μm. The smallest particle size is only 0.8 μm, while the biggest particle size can reach up to about 5.0 μm. By contrast, the Er-doped LiNi_0.5_Mn_1.5_O_4_ sample shown in Figure 2b exhibits better size distribution. Although the average particle size distribution is very close to that of the undoped LiNi_0.5_Mn_1.5_O_4_ sample, the biggest particle size has reduced to about 3.0 μm. These results indicate that the substitution of Er^3+^ ions for partial nickel and manganese ions efficiently optimize the particle size distribution and reduces the aggregation behavior to some extent, which agrees with the research results [21,22]. According to the existing literature [21], the particle size of the Er-doped LiFePO_4_ powders is less than that of undoped LiFePO_4_ and the agglomerisation of powders is decreased via Er doping in the lattice. Moreover, Xie et al. [22] has also confirmed the Er-doping can play an active role in optimizing the morphology of the Er-doped LiV_3_O_8_ sample. Figure 3a,b show the energy dispersive spectrometry (EDS) pattern of the undoped LiNi_0.5_Mn_1.5_O_4_ and Er-doped LiNi_0.5_Mn_1.5_O_4_ sample (the inset in Figure 3a,b is corresponding EDS data). According to Figure 3a,b, we can clearly observe the presence of the erbium element in the Er-doped LiNi_0.5_Mn_1.5_O_4_ sample. It should be noted that the signal strength of Er element is very small compared with Mn element, which can be attributed to the very low content of Er element. According to the ICP-OES result, the real compositions of the Er-doped LiNi_0.5_Mn_1.5_O_4_ sample is Li_0.997_Ni_0.494_Mn_1.497_Er_0.009_O_4.000_. Such low content can help explain why the signal strength of Er element is very small. The corresponding EDS data of these two samples in Figure 3a,b also illuminates the low content of Er element. Moreover, the elemental mapping images of Ni, Mn, Er, and O elements of this material are shown in Figure 3c–f. It can be seen that the erbium element is evenly distributed in the Er-doped LiNi_0.5_Mn_1.5_O_4_ sample. The above results indicate that the Er-doping can play a significant role in the optimization of product size distribution. The obtained Er-doped LiNi_0.5_Mn_1.5_O_4_ sample may show excellent electrochemical properties.

**Figure 4:**
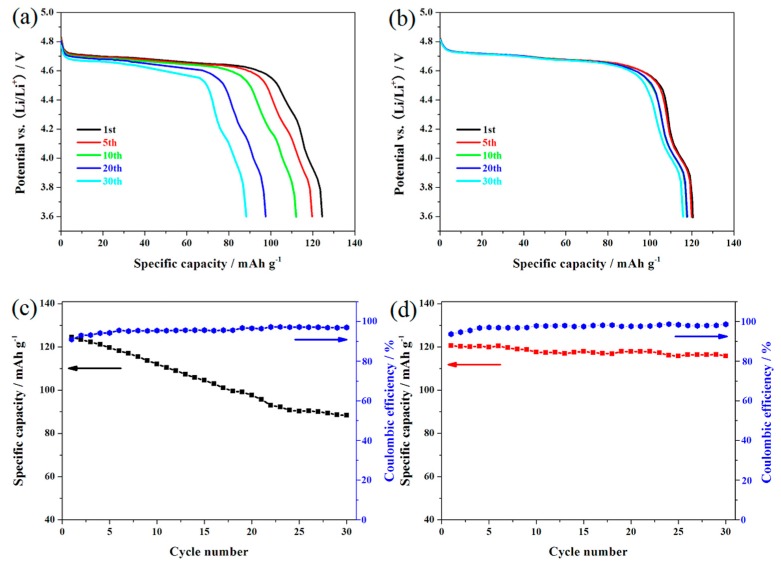
Representative charge/discharge curves of the LiNi_0.5_Mn_1.5_O_4_ (**a**) and Er-doped LiNi_0.5_Mn_1.5_O_4_ (**b**); cycling performance and coulombic efficiency of the LiNi_0.5_Mn_1.5_O_4_ (**c**) and Er-doped LiNi_0.5_Mn_1.__5_O_4_ (**d**).

To compare the effect of doping with Er^3+^ ions on the cycling stability, the LiNi_0.5_Mn_1.5_O_4_ and Er-doped LiNi_0.5_Mn_1.5_O_4_ samples were cycled at 0.5 C between 3.6 and 4.9 V. Figure 4a,b shows the representative charge/discharge curves of these two materials. As shown here, the charge/discharge curves of these two samples have a high similarity in the shape of these curves, suggesting that the substitution of Er^3+^ ions for partial nickel and manganese ions does not produce a fundamental impact on the insertion/deinsertion process of lithium ions. The Er-doped LiNi_0.5_Mn_1.5_O_4_ sample presents two obvious platforms at around 4.7 V and 4.0 V, which agrees well with that of disordered LiNi_0.5_Mn_1.5_O_4_ in the literatures [26,27]. These two plateaus can be ascribed to the redox reaction of Ni^2+/4+^ and Mn^3+/4+^, respectively. Figure 4c,d shows the cycling performances of these two materials. According to Figure 4d, the initial discharge capacity of the Er-doped LiNi_0.5_Mn_1.5_O_4_ sample is 120.6 mAh·g^−1^, which is slightly lower than that of the undoped LiNi_0.5_Mn_1.5_O_4_ sample (124.5 mAh·g^−1^). However, it is important to note that the initial discharge capacity of the undoped LiNi_0.5_Mn_1.5_O_4_ sample begins to fade greatly with the cycling going on. After 30 cycles, the Er-doped LiNi_0.5_Mn_1.5_O_4_ sample present excellent capacity retention of 93.2%. By contrast, the undoped sample shows very low capacity retention (64.5%). These results indicate that the Er-doping effectively promote the improvement of cycling stability.

**Figure 5:**
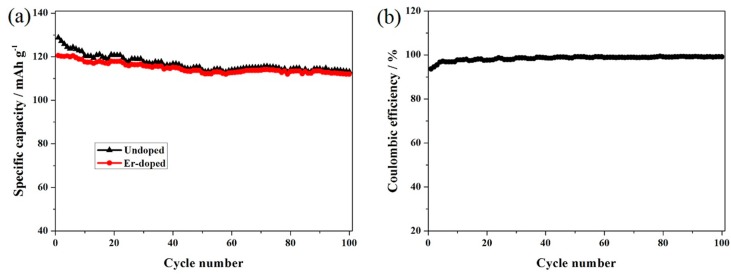
(**a**) Long cycling performance and (**b**) coulombic efficiency of the Er-doped LiNi_0.5_Mn_1.5_O_4_ at 0.5 C.

In order to investigate the cycling stability, the long cycling performance of the Er-doped LiNi_0.5_Mn_1.5_O_4_ sample was carried out at 0.5 C, as shown in Figure 5a. The Er-doped LiNi_0.5_Mn_1.5_O_4_ sample still achieves more than 112.0 mAh·g^−1^ and the capacity retention of this sample reach up to 92.9% after 100 cycles. By contrast, the cycling stability of the undoped LiNi_0.5_Mn_1.5_O_4_ sample is much worse. It delivers the bad capacity retention of 70.9% after only 30 cycles. The excellent cycling stability of the Er-doped LiNi_0.5_Mn_1.5_O_4_ sample is linked most strongly with the substitution of Er^3+^ ions for partial nickel and manganese ions in the spinel structure. For the Er-doped sample, the bonding energy of Er-O (615 kJ·mol^−1^) is higher than that of Mn-O (402 kJ·mol^−1^) and Ni-O (392 kJ·mol^−1^), which can make the spinel structure of the Er-doped LiNi_0.5_Mn_1.5_O_4_ sample become more stable [9,20,26]. Figure 5b shows the coulombic efficiency of the Er-doped LiNi_0.5_Mn_1.5_O_4_ sample after 100 cycles. As shown here, the coulombic efficiency of the initial cycle is about 93.6%, which can be explained by the side reactions of electrolyte and electrode working at high voltage [28]. As the increase of the charge/discharge cycle, the coulombic efficiency is increased to 95.4% after 3 cycles and 98.6% after 30 cycles. Such improved cycling efficiency can be ascribed to the stable solid electrolyte interphase (SEI) on the spinel after the initial cycle, which can hinder the further side reactions [29].

**Figure 6:**
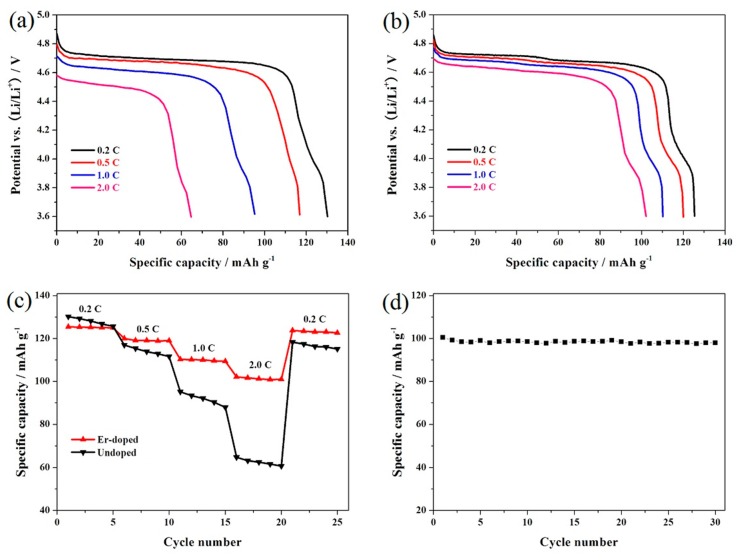
Representative discharge curves of the LiNi_0.5_Mn_1.5_O_4_ (**a**) and Er-doped LiNi_0.5_Mn_1.5_O_4_ (**b**) at different rates; (**c**) rate capability of the LiNi_0.5_Mn_1.5_O_4_ and Er-doped LiNi_0.5_Mn_1.5_O_4_; (**d**) cycling stability of the Er-doped LiNi_0.5_Mn_1.5_O_4_ at 2.0 C.

Figure 6a,b shows the representative discharge curves of the LiNi_0.5_Mn_1.5_O_4_ and Er-doped LiNi_0.5_Mn_1.5_O_4_ samples at different rates. According to Figure 6a, the undoped LiNi_0.5_Mn_1.5_O_4_ sample presents a discharge capacity loss from 130.2 mAh·g^−1^ at 0.2 C to 64.7 mAh·g^−1^ at 2.0 C along with the obvious decrement of the discharge voltage plateaus. By contrast, the Er-doping significantly enhances the rate performance of the Er-doped LiNi_0.5_Mn_1.5_O_4_. As shown in Figure 6b, the increased charge/discharge rate does not noticeably alter the representative discharge curve of the Er-doped LiNi_0.5_Mn_1.5_O_4_ sample. However, the discharge voltage plateaus of the spinel gradually decrease as the charge/discharge rate increases, which may be explained by the following two reasons. On the one hand, the higher rate can spur the increase in the polarization of cathode material, which will decrease the discharge voltage plateaus [18]. On the other hand, the increased essential resistance can cause an obvious voltage drop when lithium-ion battery is cycled at higher rate [30].

Figure 6c shows the cycling stability of the LiNi_0.5_Mn_1.5_O_4_ and Er-doped LiNi_0.5_Mn_1.5_O_4_ samples at different rates from 0.2 C to 2.0 C. As might be expected, the increase of discharge rate decreases the discharge capacity for both samples due to the limited lithium ions diffusion [31]. The Er-doped LiNi_0.5_Mn_1.5_O_4_ sample shows more prominent rate capability, especially the higher rate performance. When cycled at 0.2 C, the Er-doped LiNi_0.5_Mn_1.5_O_4_ sample present 125.4 mAh·g^−1^. As the charge/discharge rate increases to 2.0 C, the discharge capacity of this material can still retain 102.1 mAh·g^−1^, which is 81.4% of its initial discharge capacity cycled at 0.2 C. However, the undoped LiNi_0.5_Mn_1.5_O_4_ spinel only retains 49.7% at 2.0 C. In addition, when the rate restores to 0.2 C, the Er-doped LiNi_0.5_Mn_1.5_O_4_ sample delivers 123.7 mAh·g^−1^ with excellent recovery rate. Figure 6d shows the cycling stability of the Er-doped LiNi_0.5_Mn_1.5_O_4_ sample at 2.0 C. it can be seen that the Er-doped LiNi_0.5_Mn_1.5_O_4_ sample exhibits an initial discharge capacity of 100.4 mAh·g^−1^, and the capacity retention of this sample reached up to 97.6% after 30 cycles. The analysis results once again prove that the electrochemical performance of spinel LiNi_0.5_Mn_1.5_O_4_ can be greatly enhanced by the addition of Er^3+^ ions.

**Figure 7:**
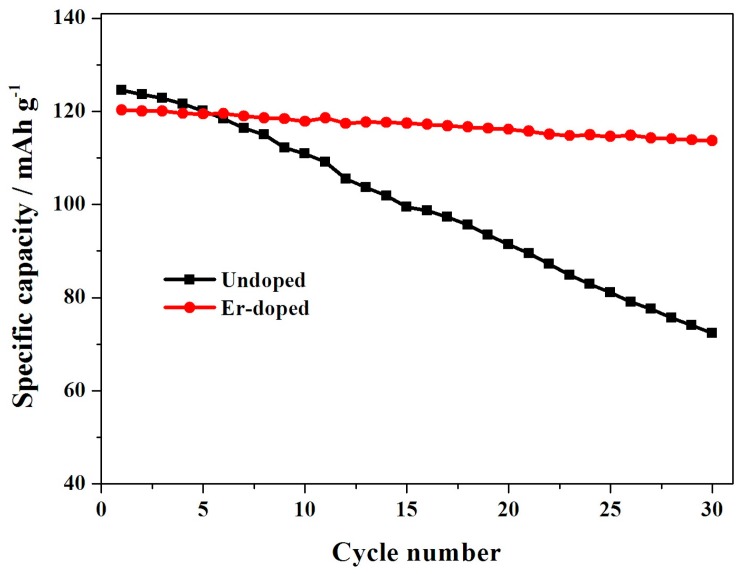
Cycling performance of the LiNi_0.5_Mn_1.5_O_4_ and Er-doped LiNi_0.5_Mn_1.5_O_4_ at 55 °C.

**Figure 8:**
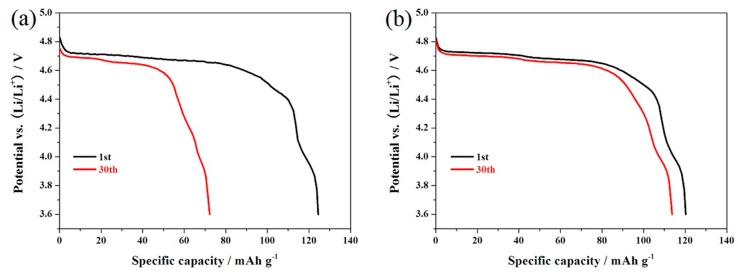
Representative discharge curves of the LiNi_0.5_Mn_1.5_O_4_ (**a**) and Er-doped LiNi_0.5_Mn_1.5_O_4_ (**b**) at 55 °C.

Figure 7 shows the cycling performance of the LiNi_0.5_Mn_1.5_O_4_ and Er-doped LiNi_0.5_Mn_1.5_O_4_ samples at 55 °C. When cycled at 0.5 C, the Er-doped LiNi_0.5_Mn_1.5_O_4_ sample exhibits an initial discharge capacity of 120.3 mAh·g^−1^, which is slightly lower than that of the undoped LiNi_0.5_Mn_1.5_O_4_ sample (124.3 mAh·g^−1^). However, after 30 cycles, the Er-doped LiNi_0.5_Mn_1.5_O_4_ sample still delivers 113.7 mAh·g^−1^, retaining 94.5% of its initial discharge capacity. Contemporaneously, the undoped LiNi_0.5_Mn_1.5_O_4_ sample retains just 58.1%. Figure 8a,b shows the representative discharge curves of the LiNi_0.5_Mn_1.5_O_4_ and Er-doped LiNi_0.5_Mn_1.5_O_4_ at 55 °C. The initial discharge curves of these two samples have a high similarity in the shape of these curves, but the corresponding 30th discharge curves present obvious difference. For the Er-doped LiNi_0.5_Mn_1.5_O_4_ sample, there is no discharge voltage drop after 30 cycles, while the discharge voltage drop of the undoped LiNi_0.5_Mn_1.5_O_4_ sample is quite obvious. The above result shows that the addition of Er^3+^ can enhance the cycling stability of LiNi_0.5_Mn_1.5_O_4_ at elevated-temperature.

**Figure 9:**
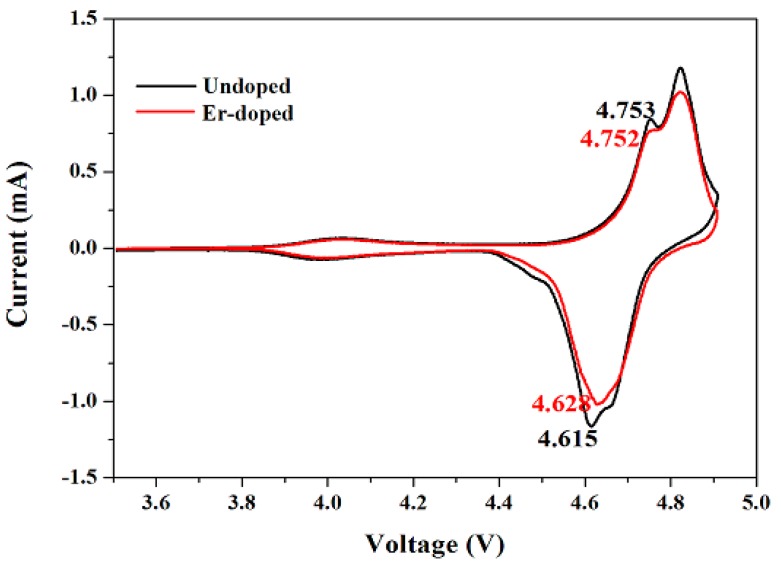
Cyclic voltammograms of the LiNi_0.5_Mn_1.5_O_4_ and Er-doped LiNi_0.5_Mn_1.5_O_4_ in the range of 3.5–4.9 V.

Figure 9 shows the cyclic voltammograms of the LiNi_0.5_Mn_1.5_O_4_ and Er-doped LiNi_0.5_Mn_1.5_O_4_ samples. The scan rate is 0.1 mV·s^−1^ and the scanning voltage is from 3.5 to 4.9 V. It can be clearly observed that these two samples present three pairs of reduction and oxidation peaks. Among them, the small reduction and oxidation peaks at around 4.0 V suggests the redox reaction of Mn^3+/4+^ couples [32]. And the main two pairs of redox peaks at around 4.7 V are ascribed to redox reactions of Ni^2+/3+^ and Ni^3+/4+^, respectively [33]. From the redox peaks of Ni^2+/3+^ of the two samples, we can find that the Er-doped LiNi_0.5_Mn_1.5_O_4_ sample presents a little smaller anodic peak and a much higher cathodic peak than the undoped LiNi_0.5_Mn_1.5_O_4_ sample. Such much smaller difference suggests a higher reversibility of electrode reaction [34].

**Figure 10:**
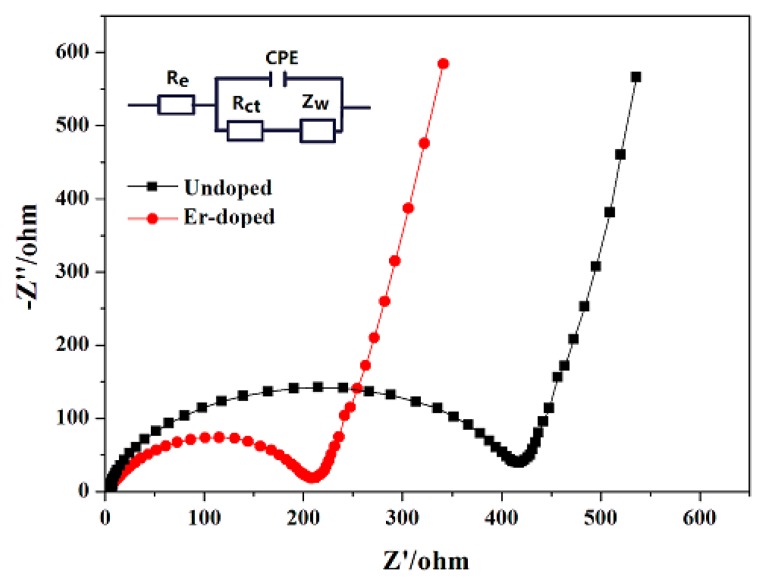
Nyquist plots of the LiNi_0.5_Mn_1.5_O_4_ and Er-doped LiNi_0.5_Mn_1.5_O_4_ before cycles (the insert is the equivalent circuit model of EIS).

Figure 10 shows the Nyquist plots of the the LiNi_0.5_Mn_1.5_O_4_ and Er-doped LiNi_0.5_Mn_1.5_O_4_ samples. The possible equivalent circuit model is embedded in Figure 10, where *R*_e_ and *R*_ct_ represent the ohmic resistance and charge transfer resistance, respectively [35,36]. And the fitting results of impedance parameters are listed in Table 1. Between the two kinds of resistances, the charge transfer resistance is much related to electrochemical property of electrode material [37]. The undoped LiNi_0.5_Mn_1.5_O_4_ sample displays a high charge transfer resistance of 377.1 Ω. However, after the addition of Er^3+^, the charge transfer resistance is decreased to 210.8 Ω, which makes it much easier for lithium-ion diffusion during the process of charge/discharge. Therefore, it should make sense why the Er-doped LiNi_0.5_Mn_1.5_O_4_ spinel presents better electrochemical property.

## 4. Conclusions

The Er-doped LiNi_0.5_Mn_1.5_O_4_ sample was successfully prepared by the citric acid-assisted sol-gel method with erbium oxide as erbium source for the first time. XRD and SEM characterization indicated that the substitution of Er^3+^ ions for partial nickel and manganese ions did not change the intrinsic structure of LiNi_0.5_Mn_1.5_O_4_ and the Er-doped LiNi_0.5_Mn_1.5_O_4_ sample showed better size distribution and regular octahedral morphology. When cycled at 0.5 C, the Er-doped LiNi_0.5_Mn_1.5_O_4_ sample exhibited an initial discharge capacity of 120.6 mAh·g^−1^, and the capacity retention of this sample reached up to 92.9% after 100 cycles. Furthermore, it could show excellent recovery rate, superior rate capability, and good high-temperature performance. The above results indicate that the substitution of Er^3+^ ions for partial nickel and manganese ions can play an active role in modifying LiNi_0.5_Mn_1.5_O_4_.

## Figures and Tables

**Table 1:** Impedance fitted data from EIS spectra for the LiNi_0.5_Mn_1.5_O_4_ and Er-doped LiNi_0.5_Mn_1.5_O_4_.

Sample	*R*_e_ (Ω)	*R*_ct_ (Ω)
LiNi_0.5_Mn_1.5_O_4_	4.01	377.13
Er-doped LiNi_0.5_Mn_1.5_O_4_	3.04	210.81

## References

[1] Fergus J.W. (2010). Recent developments in cathode materials for lithium ion batteries. J. Power Sources.

[2] Cheng J., Li X., Wang Z., Guo H. (2016). Hydrothermal synthesis of LiNi_0.5_Mn_1.5_O_4_ sphere and its performance as high-voltage cathode material for lithium ion batteries. Ceram. Int..

[3] Sulochana A., Thirunakaran R., Sivashanmugam A., Gopukumar S., Yamkiet J. (2008). Sol-gel synthesis of 5 V LiCu*_x_*Mn_2−*x*_O_4_ as a cathode material for lithium rechargeable batteries. J. Electrochem. Soc..

[4] Wei Y.J., Yan L.Y., Wang C.Z., Xu X.G., Wu F., Chen G. (2004). Effect of Ni doping on [MnO_6_] octahedron in LiMn_2_O_4_. J. Phys. Chem. B.

[5] Shigemura H., Sakaebe H., Kageyama H., Kobayashi H., West A.R., Kanno R., Morimoto S., Nasu S., Tabuchi M. (2001). Structure and electrochemical properties of LiFe*_x_*Mn_2−*x*_O_4_ (0 ≤ *x* ≤ 0.5) spinel as 5 V electrode material for lithium batteries. J. Electrochem. Soc..

[6] Mandal S., Rojas R.M., Amarilla J.M., Calle P., Kosova N.V., Anufrienko V.F., Rojo J.M. (2002). High temperature co-doped LiMn_2_O_4_-based spinels. structural, electrical, and electrochemical characterization. Chem. Mater..

[7] Sigala C., Guyomard D., Verbaere A., Piffard Y., Tournoux M. (1995). Positive electrode materials with high operating voltage for lithium batteries: LiCr*_y_*Mn_2−*y*_O_4_ (0 ≤ *y* ≤1). Solid State Ionics.

[8] Kanamura K., Hoshikawa W., Umegaki T. (2002). Electrochemical characteristics of LiNi_0.5_Mn_1.5_O_4_ cathodes with Ti or Al current collectors. J. Electrochem. Soc..

[9] Yi T.F., Zhu Y.R., Zhu R.S. (2008). Density functional theory study of lithium intercalation for 5 V LiNi_0.5_Mn_1.5_O_4_ cathode materials. Solid State Ionics.

[10] Bae S.Y., Shin W.K., Kim D.W. (2014). Protective organic additives for high voltage LiNi_0.5_Mn_1.5_O_4_ cathode materials. Electrochim. Acta.

[11] Idemoto Y., Narai H., Koura N. (2003). Crystal structure and cathode performance dependence on oxygen content of LiMn_1.5_Ni_0.5_O_4_ as a cathode material for secondary lithium batteries. J. Power Sources.

[12] Kunduraci M., Amatucci G.G. (2006). Synthesis and characterization of nanostructured 4.7 V Li*_x_*Mn_1.5_Ni_0.5_O_4_ spinels for high-power lithium-ion batteries. J. Electrochem. Soc..

[13] Kunduraci M., Al-Sharab J.F., Amatucci G.G. (2006). High-power nanostructured LiMn_2−*x*_Ni*_x_*O_4_ high-voltage lithium-ion battery electrode materials: Electrochemical impact of electronic conductivity and morphology. Chem. Mater..

[14] Ma X.H., Kang B., Ceder G. (2010). High rate micron-sized ordered LiNi_0.5_Mn_1.5_O_4_. J. Electrochem. Soc..

[15] Yoon T., Park S., Mun J., Ji H.R., Choi W. (2012). Failure mechanisms of LiNi_0.5_Mn_1.5_O_4_ electrode at elevated temperature. J. Power Sources.

[16] Jin Y.C., Lin C.Y., Duh J.G. (2012). Improving rate capability of high potential LiNi_0.5_Mn_1.5_O_4−*x*_ cathode materials via increasing oxygen non-stoichiometries. Electrochim. Acta.

[17] Xu W., Chen X., Ding F., Xiao J., Wang D.Y., Pan A.Q., Zheng J.M., Li X.H.S., Padmaperuma A.B., Zhang J.G. (2012). Reinvestigation on the state-of-the-art nonaqueous carbonate electrolytes for 5 V Li-ion battery applications. J. Power Sources.

[18] Zhao H.Y., Liu X.Q., Cheng C., Li Q., Zhang Z., Wu Y., Chen B., Xiong W. (2015). Synthesis and electrochemical characterizations of spinel LiMn_1.94_MO_4_ (M = Mn_0.06_, Mg_0.06_, Si_0.06_, (Mg_0.03_Si_0.03_)) compounds as cathode materials for lithium-ion batteries. J. Power Sources.

[19] Aklalouch M., Amarilla J.M., Rojas R.M., Saadoune I., Rojo J.M. (2008). Chromium doping as a new approach to improve the cycling performance at high temperature of 5 V LiNi_0.5_Mn_1.5_O_4_-based positive electrode. J. Power Sources.

[20] Liu H.W., Zhang K.L. (2004). The synthesis and cycling behavior of LiEr_x_Mn_2−x_O_4_ for lithium-ion batteries. Mater. Lett..

[21] Göktepe H., Sahan H., Ülgen A., Patat S. (2011). Synthesis and electrochemical properties of carbon-mixed LiE_r0.02_Fe_0.98_PO_4_ cathode material for lithium-ion batteries. J. Mater. Sci. Technol..

[22] Xie L.L., Xu Y.D., Zhang J.J., Zhang C., Cao X., Qu L. (2013). Rheological phase synthesis of Er-doped LiV_3_O_8_ as electroactive material for a cathode of secondary lithium storage. Electron. Mater. Lett..

[23] Dong Y., Young B.T., Zhang Y., Yoon T., Heskett D.R., Hu Y., Lucht B.L. (2017). Effect of lithium borate additives on cathode film formation in LiNi_0.5_Mn_1.5_O_4_/Li cells. ACS Appl. Mater. Interfaces.

[24] Chung H.T., Myung S.T., Cho T.H., Son J.T. (2001). Lattice parameter as a measure of electrochemical properties of LiMn_2_O_4_. J. Power Sources.

[25] Feng F., Liang C., Fang H., Yang B., Ma W., Dai Y. (2016). Chloride-promoted formation of octahedral LiNi_0.5_Mn_1.5_O_4_ crystal with greatly enhanced electrochemical performance. Ceram. Int..

[26] Mo M., Hui K., Hong X., Guo J., Ye C., Li A., Hu N., Huang Z., Jiang J., Liang J. (2014). Improved cycling and rate performance of Sm-doped LiNi_0.5_Mn_1.5_O_4_ cathode materials for 5 V lithium ion batteries. Appl. Surf. Sci..

[27] Arunkumar T.A., Manthiram A. (2005). Influence of chromium doping on the electrochemical performance of the 5 V spinel cathode LiMn_1.5_Ni_0.5_O_4_. Electrochim. Acta.

[28] Wu H.M., Tu J.P., Yuan Y.F., Li Y., Zhao X.B., Cao G.S. (2005). Electrochemical and ex situ XRD studies of a Li Mn_1.5_Ni_0.5_O_4_ high-voltage cathode material. Electrochim. Acta.

[29] Wen J.W., Zhang D.W., Zhang Y., Sun X., Cheng B., Ding C.X., Yu Y., Chen C.H. (2014). One-step synthesis and effect of heat-treatment on the structure and electrochemical properties of LiNi_0.5_Mn_1.5_O_4_ cathode material for lithium-ion batteries. Electrochim. Acta.

[30] Li B., Wang Y., Tu W., Wang Z., Xi M., Xing L., Li W. (2014). Improving cyclic stability of lithium nickel manganese oxide cathode for high voltage lithium ion battery by modifying electrode/electrolyte interface with electrolyte additive. Electrochim. Acta.

[31] Yi T.F., Xie Y., Zhu Y.R., Zhu R.S., Ye M.F. (2012). High rate micron-sized niobium-doped LiMn_1.5_Ni_0.5_O_4_ as ultra high power positive-electrode material for lithium-ion batteries. J. Power Sources.

[32] Ding Y.L., Xie J., Cao G.S., Zhu T.J., Yu H.M., Zhao X.B. (2011). Single-crystalline LiMn_2_O_4_ nanotubes synthesized via template-engaged reaction as cathodes for high-power lithium ion batteries. Adv. Funct. Mater..

[33] Xiong L.L., Xu Y.L., Tao T., Goodenough J.B. (2012). Synthesis and electrochemical characterization of multi-cations doped spinel LiMn_2_O_4_ used for lithium ion batteries. J. Power Sources.

[34] Kunduraci M., Amatucci G.G. (2007). Effect of oxygen non-stoichiometry and temperature on cation ordering in LiMn_2−*x*_Ni*_x_*O_4_ (0.50 ≥ *x* ≥ 0.36) spinels. J. Power Sources.

[35] Markovsky B., Talyossef Y., Salitra G., Aurbach D., Kim H.J., Choi S. (2004). Cycling and storage performance at elevated temperatures of LiNi_0.5_Mn_1.5_O_4_ positive electrodes for advanced 5 V Li-ion batteries. Electrochem. Commun..

[36] Zhong Q., Bonakdarpour A., Zhang M., Gao Y., Dahn J.R. (1997). Synthesis and electrochemistry of LiNi*_x_*Mn_2−*x*_O_4_. J. Eletrochem. Soc..

[37] Feng C.Q., Li H., Zhang C.F., Guo Z.P. (2012). Synthesis and electrochemical properties of non-stoichiometric Li-Mn-spinel (Li_1.02_M*_x_*Mn_1.95_O_4−*y*_F*_y_*) for lithium ion battery application. Electrochim. Acta.

